# Weak protein–bicelle binding quantification via surface-based DNA nanolevers

**DOI:** 10.1007/s00249-026-01829-4

**Published:** 2026-03-02

**Authors:** Sophie Combet, Raphael Dos Santos Morais, Audrey Comte, Angélique Cheron, Emeline Barbet-Massin, Hanna Müller-Landau, Olivier Delalande, Jean-François Hubert, Jean-Baptiste Charbonnier, Paloma Fernández Varela

**Affiliations:** 1 Laboratoire Léon-Brillouin (LLB), UMR12 CEA, CNRS, Université Paris-Saclay, Gif-sur-Yvette CEDEX, F-91191 France; 2 Université de Rennes, CNRS, IGDR, UMR6290, Rennes, F-35000 France; 3 Institute for Integrative Biology of the Cell (I2BC), CEA, CNRS, Université Paris-Saclay, Gif-sur-Yvette CEDEX, F-91198 France; 4 Dynamic Biosensors GmbH, Perchtinger Str. 8/10, D-81379 München, Germany; 5 JEOL Germany GmbH, Gute Änger 30, D-85356 Freising, Germany; 6 Quattro research, Fraunhoferstrasse 18a, Planegg-Martinsried, D-82152 Germany

**Keywords:** Bicelles, Binding kinetics, Biosensor technology, Dystrophin, Protein-lipid interactions, Weak affinities

## Abstract

To investigate how human dystrophin associates with biomimetic phospholipid bicelles, we applied switchSENSE^®^, a fluorescence-based biosensing technology that employs dynamic DNA nanolevers in a microfluidic environment with minimal material requirements. We show that switchSENSE^®^ enables real-time kinetic analysis of weak and transient interactions, offering a clear advantage over classical biophysical methods that often fail to capture low-affinity protein-lipid binding. Focusing on two functionally relevant central-domain dystrophin fragments (R1-3 and R11-15), we quantified binding to zwitterionic and anionic bicelles using two complementary assay orientations: proteins immobilized with bicelles as analytes, and the reverse. Both yielded consistent micromolar affinities, in line with prior microscale thermophoresis results, but uniquely provided kinetic rate constants. This represents the first kinetic characterization of dystrophin-bicelle interactions, revealing that weak affinity is largely driven by rapid dissociation. Notably, R11-15 exhibited faster association and slower dissociation than R1-3, indicating a more stable and sustained lipid interaction. Beyond advancing mechanistic insight into dystrophin’s reversible membrane association – a process thought to stabilize sarcolemmal integrity during muscle contraction and elongation -, our study highlights switchSENSE^®^ as a versatile platform for quantifying weak protein-lipid interactions. By enabling kinetic resolution of interactions at the edge of conventional detection limits, switchSENSE^®^ provides a powerful tool for dissecting the dynamic interplay between structural proteins and lipid assemblies.

**Supplementary Information:**

The online version contains supplementary material available at 10.1007/s00249-026-01829-4.

## Introduction

Protein–protein and protein–lipid interactions are fundamental to virtually all cellular processes, and their quantitative characterization is essential to understand biological functions. Many physiologically relevant interactions - such as those involved in cell adhesion (van der Merwe and Barclay 1994; Vaynberg and Qin 2006), fragment-based drug design (Mendes and Blundell 2017; Murray and Rees 2009), and liquid–liquid phase separation (Giudice and Jiang 2024; Khorsand and Uversky 2024; Mukherjee et al. 2024; Zhang et al. 2023) - are intrinsically weak, with dissociation constants (*K*_*d*_) in the micromolar (µM) to ~ 100 µM range. Such low-affinity interactions often evade detection by conventional biophysical techniques.

Standard methods such as surface plasmon resonance (SPR) or fluorescence anisotropy are well suited for strong interactions (*K*_*d*_ < 1 µM) but generally lack the sensitivity to detect weaker systems. ITC can be applied to quantify low-affinity interactions under suitable experimental conditions; however, the main challenge remains the stability of the highly concentrated samples needed for reliable measurements.

More specialized approaches – including nuclear magnetic resonance (NMR) (Vaynberg and Qin 2006; Xu et al. 2024), analytical ultracentrifugation (Fedorov et al. 2020), and microscale thermophoresis (MST) (Mias-Lucquin et al. 2020; Dos Santos Morais et al. 2018) – have proven useful in this context. In our previous work, we used MST to detect for the first time the weak interactions between two central-domain dystrophin fragments (R1-3 and R11-15) and phospholipid bicelles (Mias-Lucquin et al. 2020; Dos Santos Morais et al. 2018), although this method did not provide access to the kinetic constants.

Here, we extend this line of investigation by applying switchSENSE^®^ (Langer et al. 2013; Knezevic et al. 2012), an advanced real-time biosensing technology, to quantify protein-lipid affinities and kinetics using dystrophin-bicelle binding as a model system. Although switchSENSE^®^ has been widely applied to protein-protein and protein-DNA interactions (Kefala Stavridi et al. 2023; Mak et al. 2023; Marcelot et al. 2021; Nemoz et al. 2018; Schenckbecher et al. 2021; Schiedel et al. 2020; Velours et al. 2021), this is, to our knowledge, the first report of its use with membrane lipid systems. Crucially, our study establishes switchSENSE^®^ as a powerful and versatile platform for resolving weak protein-bicelle interactions, opening new opportunities to investigate peripheral membrane proteins and other challenging biomolecular interfaces.

**Fig. 1:**
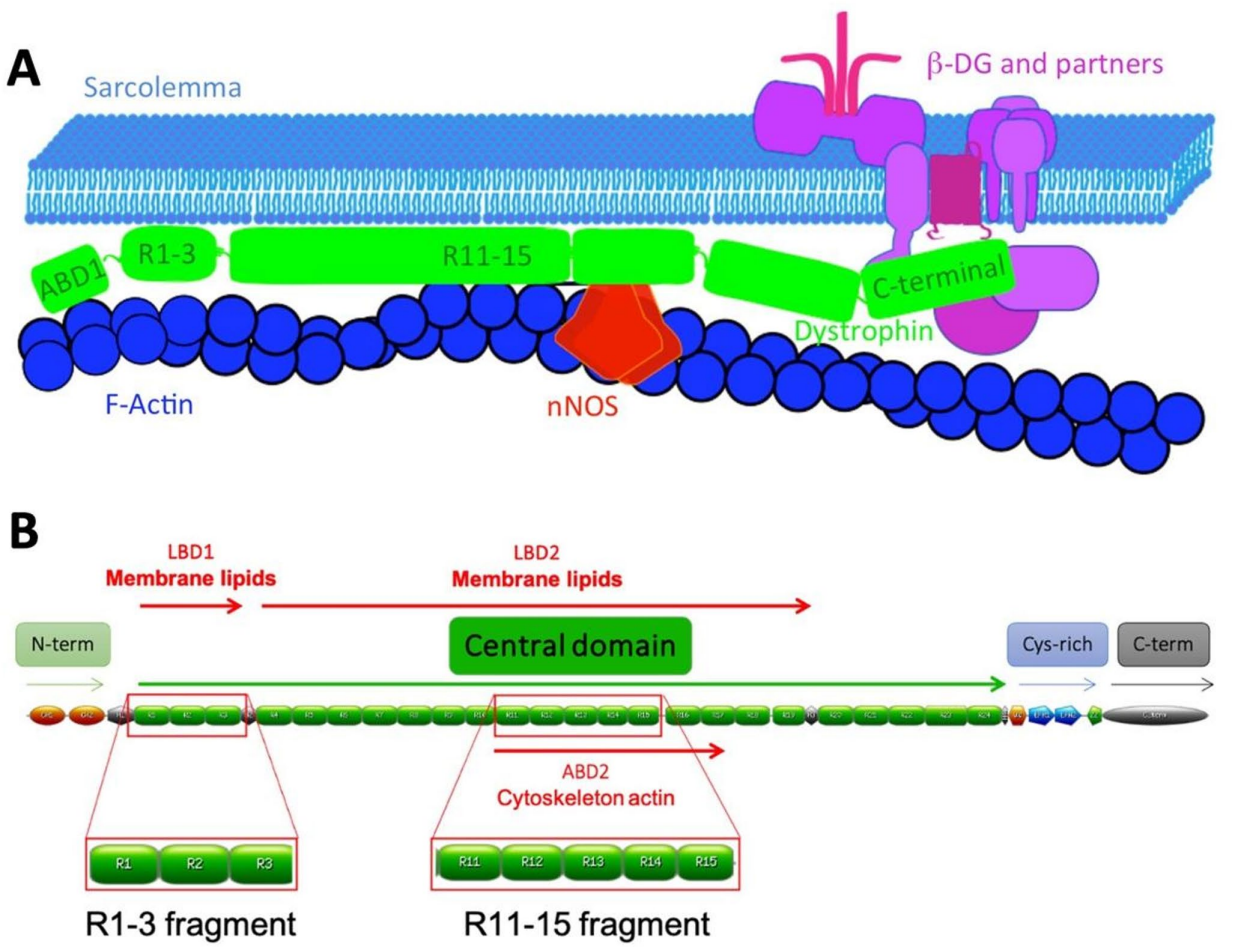
Structural organization and membrane interactions of dystrophin. (**A**) Schematic representation of the dystrophin protein (green) in muscle cells, illustrating its interactions with the sarcolemma (light blue), filamentous actin (F-actin, dark blue), neuronal nitric oxide synthase (nNOS, red), and the β-dystroglycan complex (β-DG, magenta). (**B**) Domain architecture of dystrophin’s central rod region (green), composed of 24 spectrin-like repeats. The fragments R1-3 and R11-15 analysed in this study are highlighted. Both are known to associate sarcolemma phospholipids in muscle cells

switchSENSE^®^ is a microfluidic biosensing platform developed by Dynamic Biosensors, based on fluorescently labeled DNA nanolevers tethered to gold electrodes. The system operates in two modes: (*i*) a static mode, where binding is detected via fluorescence proximity sensing, and (*ii*) a dynamic mode, where an alternating electric field drives nanolever motion. Binding events increase hydrodynamic drag, altering nanolever kinetics and providing insights into both complex formation and conformational changes. This technology offers several advantages over traditional surface-based approaches: (*i*) matrix-free surface that minimize non-specific binding; (*ii*) flexible tethers that preserve ligand mobility; (*iii*) tuneable ligand density; (*iv*) minimal material consumption due to localized fluorescence detection; and (*v*) internal referencing for signal accuracy. To date, applications have focused mainly on soluble complexes with only one related example describing an antibody-receptor interaction on virus-like particles (*Dynamic Biosensors link*). Its potential for studying membrane-associated interactions therefore remains largely unexplored. We selected dystrophin as a model peripheral membrane protein, to illustrate this new application. Dystrophin is a large filamentous protein essential for sarcolemma stability in muscle cells (Legardinier et al. 2008; Zhao et al. 2016) and mutations in its gene cause dystrophinopathies (Monaco 1989). The protein central-domain consists of 24 spectrin-like repeats arranged as triple coiled-coils (Legardinier et al. 2009) (Fig. 1). Because full-length dystrophin cannot be recombinantly produced, we focused on two functional relevant fragments, R1-3 and R11-15 (“R” from repeats), which are known to interact with membrane phospholipids both in vitro (Mias-Lucquin et al. 2020; Dos Santos Morais et al. 2018; Sarkis et al. 2013; Le Rumeur et al. 2010) and in vivo (Zhao et al. 2016). These domains are also relevant for current gene therapy strategies based on “mini-dystrophins” (Barthélémy and Wein 2018; Belanto et al. 2014; Chamberlain and Chamberlain 2017; Shieh 2018). As a biomimetic membrane model, we used isotropic phospholipid bicelles (Dos Santos Morais et al. 2017). These disk-shaped assemblies consist of bilayer-formed by relatively long-chain lipids (DMPC, DMPS) with the rim stabilized by relatively short-chain lipids (DHPC). Bicelle size is defined by the effective molar ratio *q*_*eff*_ as:

1 $${q}_{eff}=\frac{\left[DMPC\: + \:DMPS\right]}{{\left[DHPC\right]}_{total}-{\left[DHPC\right]}_{free}}$$

We employed bicelles with a *q*_*eff*_ of 1.3, corresponding to an average diameter of 10 nm, while maintaining free DHPC at 6 mM (Beaugrand et al. 2014; Dos Santos Morais et al. 2017; Dürr et al. 2012). This size matches well with dystrophin fragments maximal dimensions measured by SAXS (~ 17–23 nm) (Delalande et al. 2018) and it provides a sufficiently extended planar lipid surface for protein interaction while maintaining reproducible kinetic measurements. Compared to liposomes or cellular membranes, bicelles exhibit limited curvature, thereby minimizing curvature-induced effects and allowing us to primarily investigate lipid composition–dependent interactions. While membrane curvature can influence the binding properties of curvature-sensitive proteins, dystrophin fragments are not known to display strong curvature preference. Importantly, the use of bicelles ensures methodological continuity with our previous MST and SANS studies, enabling direct comparison across complementary biophysical techniques. Zwitterionic and anionic bicelles were selected to mimic the compositional heterogeneity of the sarcolemmal membrane, particularly the enrichment of negatively charged phospholipids such as phosphatidylserine in the inner leaflet.

In earlier work, we confirmed lipid binding of dystrophin fragments by MST and identified conformational changes of R1-3 upon bicelle binding through tryptophan fluorescence, small-angle neutron scattering (SANS), and molecular simulations (Dos Santos Morais et al. 2018). Here, we extended these studies by applying switchSENSE^®^ in two complementary configurations: immobilizing dystrophin fragments on the biochip and injecting bicelles in solution, and the reverse arrangement with tethered bicelles and dystrophin fragments as analytes. Together, these configurations validate the ability of switchSENSE^®^ to detect, quantify, and characterize weak protein–lipid interactions in different orientations.

By establishing switchSENSE^®^ as a method for studying protein-bicelle interactions, this work provides a new experimental framework for investigating peripheral membrane protein, which frequently engage in weak and transient lipid interactions.

## Materials and methods

### Protein and bicelle preparation

The R1-3 and R11-15 fragments of human dystrophin (Uniprot P11532), comprising 333 residues (38.5 kDa) and 515 residues (60 kDa), respectively, were produced in *Escherichia coli* BL21 (DE3) bacteria. Their sequences were *GS-*EVNLD.QISQA (R1-3) and *GS-*FQKPA.LNFAQ (R11-15), each carrying an N-terminal His-tag followed by a thrombin-cleavage site (italicized residues), as previously described (Dos Santos Morais et al. 2018; Mias-Lucquin et al. 2020).

Protein expression was induced with 1 mM IPTG for 4 h at 37 °C. After cell lysis, both fragments were purified by immobilized metal affinity chromatography (IMAC) on Ni-Sepharose columns (HisTrap, GE Healthcare). The His-tags were subsequently removed by thrombin cleavage, and the proteins were further purified by size-exclusion chromatography (SEC) using a HiLoad 16/600 Superdex 200 prep Grade column (GE Healthcare) equilibrated in TNE buffer (20 mM Tris, 150 mM NaCl, 0.1 mM EDTA, pH 7.5). Protein purity and molecular weights were confirmed by SDS-PAGE (Fig. S2A). Protein concentrations were determined using theoretical molar extinction coefficients at 280 nm: 59,720 M^− 1^ cm^− 1^ for R1-3 and 45,950 M^− 1^ cm^− 1^ for R11-15.

Zwitterionic bicelles (DMPC: DHPC, 1:1) and anionic bicelles (DMPC: DMPS: DHPC, 0.67:0.33:1) were prepared as previously described (Dos Santos Morais et al. 2017, 2018), at a total lipid concentration of 200 mM. These bicelles form disk-shaped assemblies of ~ 9 nm diameter and ~ 4 nm thickness, as determined by SANS (Dos Santos Morais et al. 2017). All phospholipids were purchased from Avanti Polar Lipids (Alabaster, AL, USA) and used without further purification.

### Dynamic light scattering (DLS)

The monodispersity and hydrodynamic radius (*R*_*h*_) of the dystrophin fragments were assessed using a Zetasizer Nano ZS (Malvern Instruments) at room temperature, in 100 µL low-volume quartz cuvettes (Hellma). Data were analysed with Zetasizer software v7.11, using default parameters and reporting the intensity-based size distribution. -The hydrodynamic radius (*R*_*h*_) was calculated from the diffusion coefficient using the Stokes-Einstein equation:

2 $${R}_{h}=\frac{{k}_{B}T}{6\:\pi\:\:\eta\:\:D}$$

where *k*_*B*_ is the Boltzmann constant, *T* the absolute temperature, and *η* the viscosity of the solvent.

### switchSENSE^®^ DRX^2^ device and biochips

All experiments were performed on a DRX^2^ instrument (Dynamic Biosensors GmbH) equipped with dual light sources and photon counters. For kinetic measurements, we used standard multipurpose biochips carrying 48- and 96-nucleotide DNA nanolevers (NLs), MPC2-48-2-G1R1-S, MPC-96-1-R1-S and MPC2-96-2-G1R1-S (Dynamic Biosensors GmbH). In these designations, “MPC” refers to multipurpose chips, and “S” to standard format. The numbers “48” or “96” indicate the DNA nanolever length in nucleotides. “R1” denotes chips containing a single red fluorescent probe and two DNA sequences (sequence A on electrodes 1–2 and sequence B on electrodes 3–6) (Müller-Landau and Varela 2021). “G1R1” identifies dual-colour chips, which carry both green- and red- labelled DNA sequences (sequence A, green; sequence B, red) enabling two-colour measurements across all six electrodes (Müller-Landau and Varela 2021). For sizing experiments, only 48 nucleotide DNA NLs were used.

These biochips comprise four microfluidic channels, each containing six gold microelectrodes coated with DNA NLs. The 5’ ends of the single-stranded NLs are anchored to the gold surface, while the 3’ ends are exposed and fluorescently labelled (green or red), as described above. This configuration enables simultaneous detection of two different interactions on the same electrode, through distinct fluorescent signals associated with each DNA sequence (Müller-Landau and Varela 2021).

### Sample and switchSENSE^®^ surface preparation

**Fig. 2:**
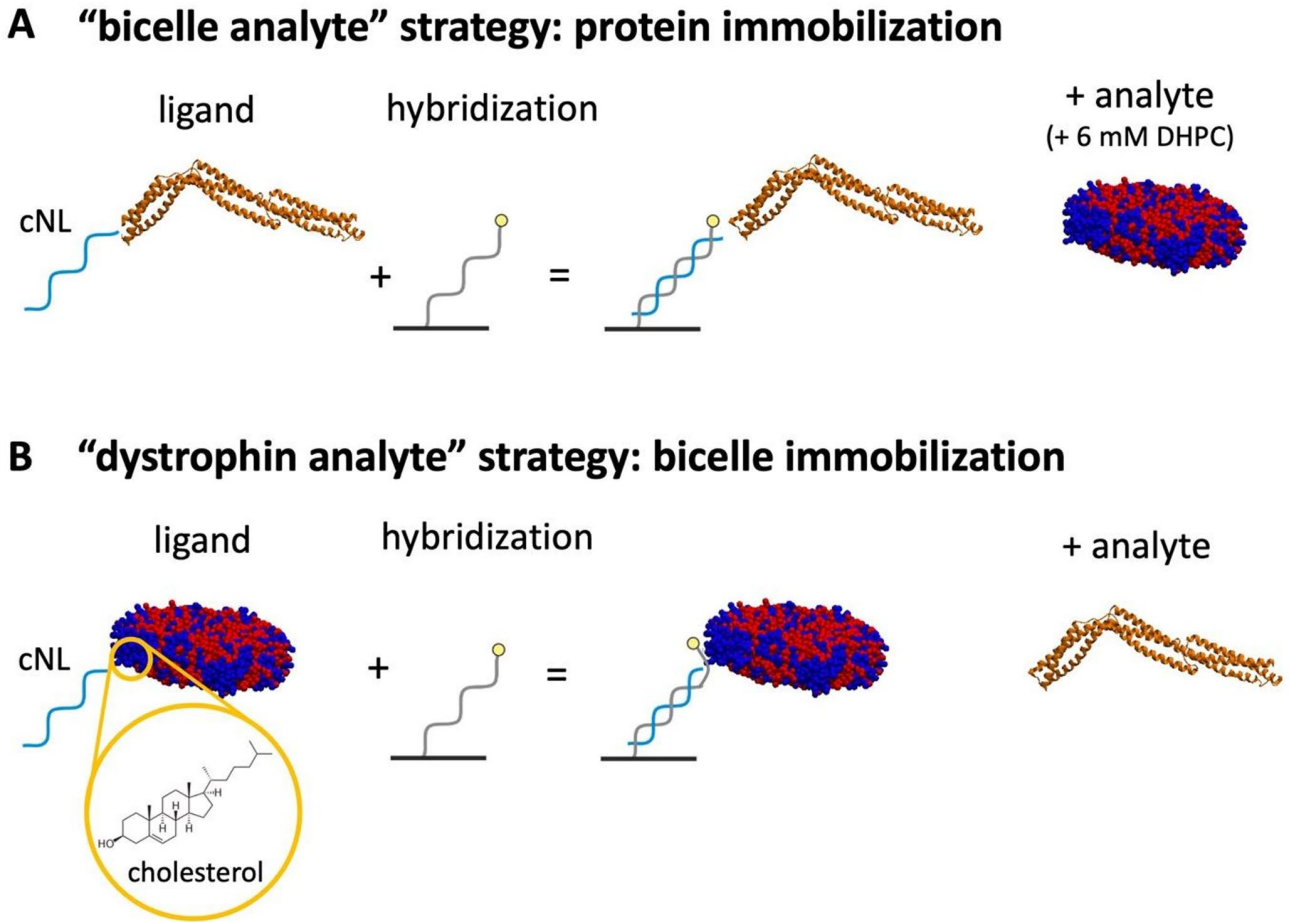
Experimental strategies for studying dystrophin-bicelle interactions using fluorescent DNA nanolevers (NLs) with switchSENSE^®^ technology. (**A**) *“Bicelle as analyte” strategy.* Dystrophin fragments (e.g. R1-3), are covalently cross-linked to complementary NLs (cNLs) and immobilized on the chip as ligands. Phospholipid bicelles - composed here of zwitterionic DMPC (red) and DHPC (blue) - are injected as analytes. (**B**) *“Dystrophin as analyte” strategy.* Cholesterol-modified cNLs capture bicelles by insertion of cholesterol into their hydrophobic parts, thereby immobilizing them on the chip surface as ligands. Dystrophin fragments (e.g. R1-3) are then injected as analytes

Immobilization of ligands on the switchSENSE^®^ chip requires a complementary DNA nanolever (cNL), either 48 or 96 nucleotides in length, designed to hybridize with the fluorescently labelled single-stranded DNA nanolever (NL) tethered to the biochip surface. The ligand-cNL conjugate is hybridized to the surface-bound NLs, thereby functionalizing the sensor surface for sizing or interaction studies (Fig. 2).

The analyte of interest then flows over the functionalized surface, and binding events between the analyte and immobilized ligand are monitored in real-time using switchSENSE^®^ technology.

In this study, two complementary strategies were employed:

i. ***“Bicelle as analyte” strategy*** (Fig. 2A). Dystrophin fragments were covalently cross-linked to cNLs complementary to the surface-immobilized NLs. Conjugation was performed using the “Amine Coupling Kit 1 for Proteins (> 5 kDa)” (Dynamic Biosensors GmbH) according to the manufacturer’s protocol. Depending on the chip, dystrophin fragments were conjugated to either 48- and 96-nucleotide cNLs. The preparation of conjugated dystrophin samples followed a protocol previously published (Müller-Landau and Varela 2021). Briefly, cNLs are activated with amine-reactive groups, followed by the addition of dystrophin fragments in molar excess to ensure complete conjugation. After ≥ 1 h incubation at room temperature, the resulting dystrophin-cNL conjugates were purified from unreacted cNL by anion exchange chromatography. An example of the R1-3-cNL purification is shown in Fig. S2B.

The purified dystrophin-cNL conjugates were hybridized to the DNA NLs on the chip, thereby immobilizing dystrophin fragments as ligands. Bicelles, acting as analytes, were then flowed across the surface to probe binding interactions.

ii. ***“Dystrophin as analyte” strategy*** (Fig. 2B). In a complementary setup, lipid bicelles were immobilized on the chip surface using cholesterol-modified cNL (48- or 96-nucleotides; https://www.biomers.net/*).* These constructs included a triethylene glycol (TEG) spacer between the DNA and cholesterol moiety to enhance accessibility for binding. The cholesterol-modified cNL were hybridized to the DNA NLs on the chip, after which bicelles were injected and intercalated with the cholesterol anchors on the surface. Dystrophin fragments, injected as analytes, were then flowed over the immobilized bicelles to monitor their interaction.

### switchSENSE^®^ sizing and binding affinity measurements

Protein hydrodynamic radius was determined using the dynamic mode of the switchSENSE^®^ DRX^2^ device (Dynamic Biosensors GmbH), which measures the influence of protein binding on the switching kinetics of double stranded DNA sensors (Müller-Landau and Varela 2021). Binding kinetics (association and dissociation rates) and affinity constants, were assessed in static mode, using fluorescence proximity sensing (FPS). In this configuration, the red probe (DNA sequence B) (Müller-Landau and Varela 2021) was functionalized with the target molecule, while the green probe (DNA sequence A) (Müller-Landau and Varela 2021) served as an internal control.

For DNA hybridization and sizing experiments, TE40 buffer (10 mM Na_2_HPO_4_/NaH_2_PO_4_, 40 mM NaCl, 0.05 % Tween 20, 50 µM EDTA, and 50 µM EGTA) was used, as the hybridization and running buffer in dynamic modes. DNA strands for cNL-B96 and cNL-B48 (the latter only for sizing experiments) were prepared according to the manufacturer’s instructions. Regeneration and passivation solutions, as well as glass vials, were obtained from Dynamic Biosensors GmbH. Protocols were designed using the *switchBUILD* software and executed with *switchCONTROL* software (Dynamic Biosensors GmbH) (Müller-Landau and Varela 2021).

To minimize non-specific binding, gold microelectrode surfaces were passivated with a thiol-containing solution prior to measurements. In dynamic mode, hydrodynamic diameters (*D*_*h*_) were determined individually for each of the six parallel electrodes, with averaged values computed from the electrodes passing quality thresholds. For kinetics assays, the electrode with the highest fluorescence amplitude (as determined by the chip status test) was used for data acquisition. Each flow channel was subjected to a maximum of four regeneration cycles, each involving ligand-analyte dissociation followed by re-hybridization with fresh cNLs.

For immobilization of commercial cNL references, a DNA concentration of 500 nM was hybridized on-chip for 320 s. For cNL constructs conjugated with dystrophin fragments or bicelles, DNA concentration was reduced to 100 nM and hybridization time was doubled to conserve samples. The running buffer for kinetics consisted of TNE (20 mM Tris, 150 mM NaCl, and 0.1 mM EDTA), supplemented with 6 mM DHPC to stabilize bicelles. For zwitterionic bicelles, association and dissociation phases were performed at 50 µL min^− 1^ for 200 s each. For anionic bicelles, the flow rate was increased to 100 µL min^− 1^, with association for 100 s and dissociation for 200 s. An identical blank run without ligand served as a reference to correct background signal. All experiments were conducted at 25 °C.

### Affinity and kinetics analysis

In surface-based biosensors, the system is simplified because the number of captured molecules on the sensor surface is fixed. When the analyte concentration in solution is much higher than the number of available binding sites, and a continuous flow of fresh solution is maintained, the analyte concentration remains effectively constant during binding.

A key parameter is the fraction bound (*fb*), representing the ratio of occupied to total binding sites, which is directly proportional to the sensor signal. The fraction bound ranges from 0% (unbound) to 100% (fully saturated surface).

Under equilibrium conditions (time$\:\to\:\infty\:$), the law of mass action yields the Langmuir isotherm, relating *fb* to analyte concentration *c* (Müller-Landau and Varela 2021) (see also Dynamic Biosensors technical note: https://www.dynamic-biosensors.com/wpcms/wp-content/uploads/2016/05/technote_101_binding-theory.pdf):

3 $${fb}_{eq}\left(c\right)=\frac{c}{c\:+\:{K}_{d}}$$

where *c* is the analyte concentration, and *K*_*d*_ is the equilibrium dissociation constant (in M), defined as *K*_*d*_ *= k*_*off*_/*k*_*on*_, with *k*_*on*_ the association rate constant (M^− 1^ s^− 1^) and *k*_*off*_ the dissociation rate constant (s^− 1^).

The interaction between ligand and analyte on the sensor surface can be described in two phases:

*Association phase (analyte present).* Upon introduction of analyte at concentration *c*, the fraction of bound ligand evolves over time as:

4 $$fb(t,c)={fb}_{eq}\left(c\right)(1-{e}^{-{k}_{on}^{obs}t})$$

where *fb*_*eq*_(*c*) is the fraction bond, $\:{k}_{on}^{obs}=c.{k}_{on}\:+{\:k}_{off}$ is the observed association rate constant, and ${\tau\:}_{on}^{obs}\:=\:\frac{1}{{k}_{on}^{obs}}$ is the association time constant.

*Dissociation phase (analyte removed).* When the analyte is replaced by buffer (*c* = 0), the decay of bound complex follows:

5 $$fb\left(t\right)=a{e}^{-{k}_{off}t}$$

where *a* is the initial bound fraction at the start of dissociation and $\:{\tau\:}_{off}=\frac{1}{{k}_{off}}$ is the dissociation time constant.

### Analysis of association and dissociation kinetics

Fluorescence data were acquired continuously throughout both association and dissociation phases, with no interruption at the transition between the two. The apparent gap visible between fitted curves does not indicate missing measurements but results from the fitting strategy. To ensure robust estimation of dissociation rate constants (*k*_off_), curve fitting was preferentially applied to all dissociation phase time points, where the signal decay more closely follows mono-exponential behaviour. Few early time points were occasionally excluded when they displayed deviations potentially arising from transient effects associated with solution exchange or instrumental stabilization, which could otherwise bias kinetic estimates.

### Graphs

All kinetics graphs were generated using Origin software.

## Results and discussion

To investigate macromolecular interactions using switchSENSE^®^ technology, the workflow comprises two main steps, as in other surface-based methods: ligand immobilization and analyte interaction measurement. A key innovation of this approach is the immobilization of ligands on the sensor surface via DNA hybridization.

Two experimental configurations were employed to explore the interaction between dystrophin fragments and bicelles. In the “bicelle analyte” setup (Fig. 2A), bicelles were flowed over immobilized dystrophin fragments to assess binding. Conversely, the “dystrophin analyte” setup (Fig. 2B) allowed investigation of dystrophin fragments binding to surface coated with bicelles.

### Dystrophin fragment sizing measurement

**Fig. 3:**
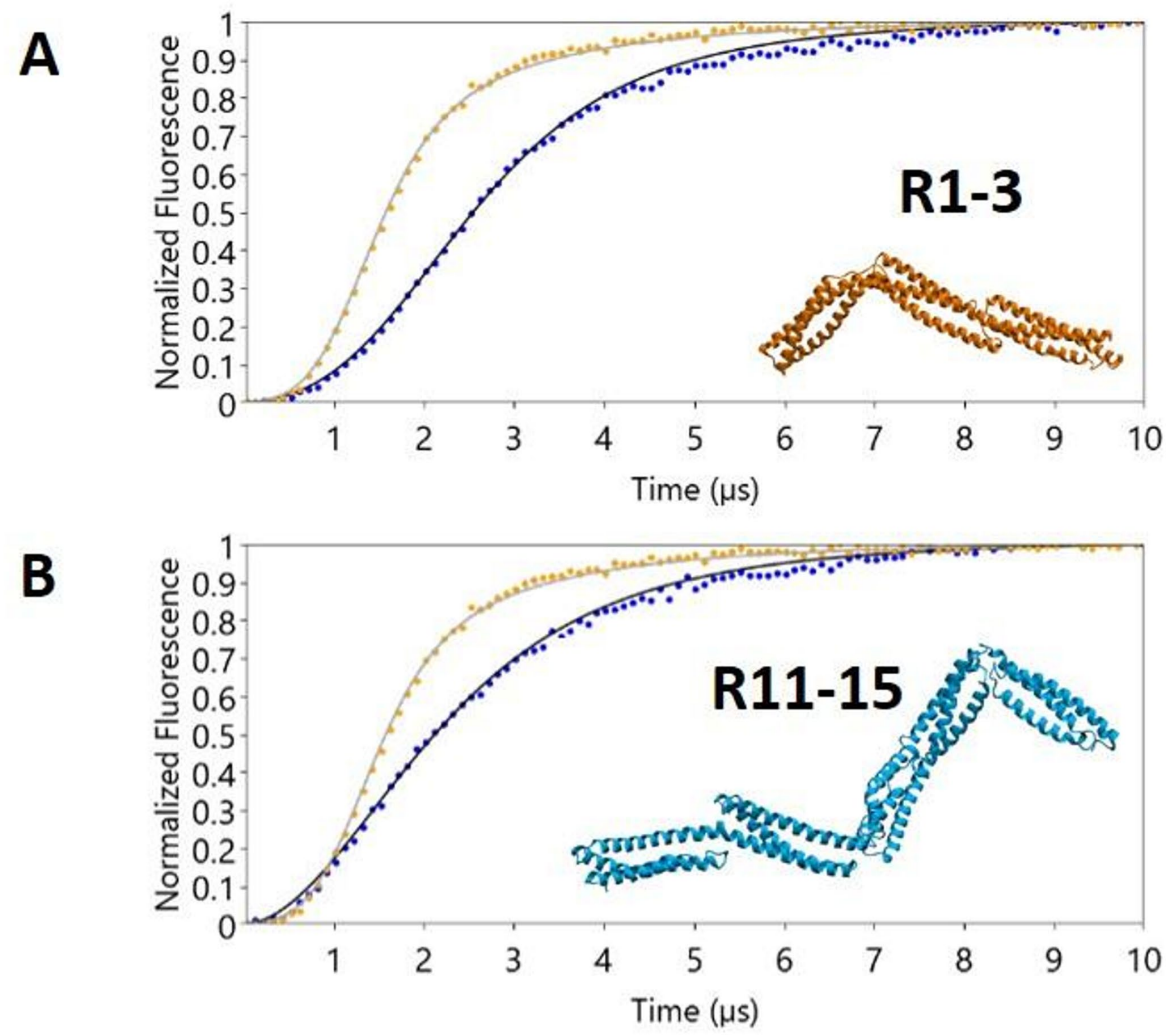
Hydrodynamic sizing of dystrophin fragments using the dynamic mode of switchSENSE^**®**^. Sizing was performed for (**A**) R1-3 and (**B**) R11-15 fragments by monitoring the normalized fluorescence response of DNA-crosslinked dystrophin over time (µs). Measurements were conducted on six electrodes in parallel. The fluorescence signal is shown in blue, with the fitted curve in black. For comparison, control measurements using reference DNA without protein are shown in orange, with the corresponding fit in grey. The reduced switching speed observed in the presence of protein (i.e. decreased slope) reflects increased hydrodynamic size, in agreement with the “lollypop” model of tethered DNA motion [13]

The hydrodynamic diameter (*D*_*h*_) of DNA-conjugated dystrophin fragments was measured using the dynamic mode of switchSENSE^®^(Fig. 3) and compared with the results obtained by dynamic light scattering (DLS). switchSENSE^®^ measurements yielded *D*_*h*_ values of 5.2 ± 0.3 nm for R1-3 and 8.1 ± 0.4 nm for R11-15 dystrophin fragments, slightly smaller than those obtained by DLS (8 ± 2 nm and 12 ± 4, respectively; Fig. 3). Despite this systematic offset, the ratio of *D*_*h*_ between the two fragments remains consistent across both techniques. Notably, the *D*_*h*_ for R11-15 also agrees with the Stokes diameter of ~ 8.2 nm previously determined by size-exclusion chromatography (SEC) supporting the reliability of the measurements (Legardinier et al. 2009).

### Kinetics of bicelle-dystrophin interactions

Kinetics were measured using the static mode of switchSENSE^®^, in which the DNA nanolevers (NLs) remain extended away from the sensor surface at a fixed angle. Data collected with the shorter 48-DNA NLs were uninterpretable due to the relatively large size of the bicelles (data not shown), so all kinetic experiments were performed using the longer 96-DNA NLs.

***“Bicelle analyte” strategy***. In this configuration, dystrophin fragments were covalently immobilized on the chip surface, enabling real-time monitoring of interactions with bicelles as analytes. Association and dissociation phases were fitted to extract the kinetic rate constants: the association rate constant *k*_*on*_ (M^− 1^ s^− 1^), the dissociation rate constant *k*_*off*_ (s^− 1^), and the equilibrium dissociation constant *K*_*d*_ = *k*_*off*_/*k*_*on*_ (M), which represents the inverse of association constant (see Eqs. 3–5).

**Fig. 4:**
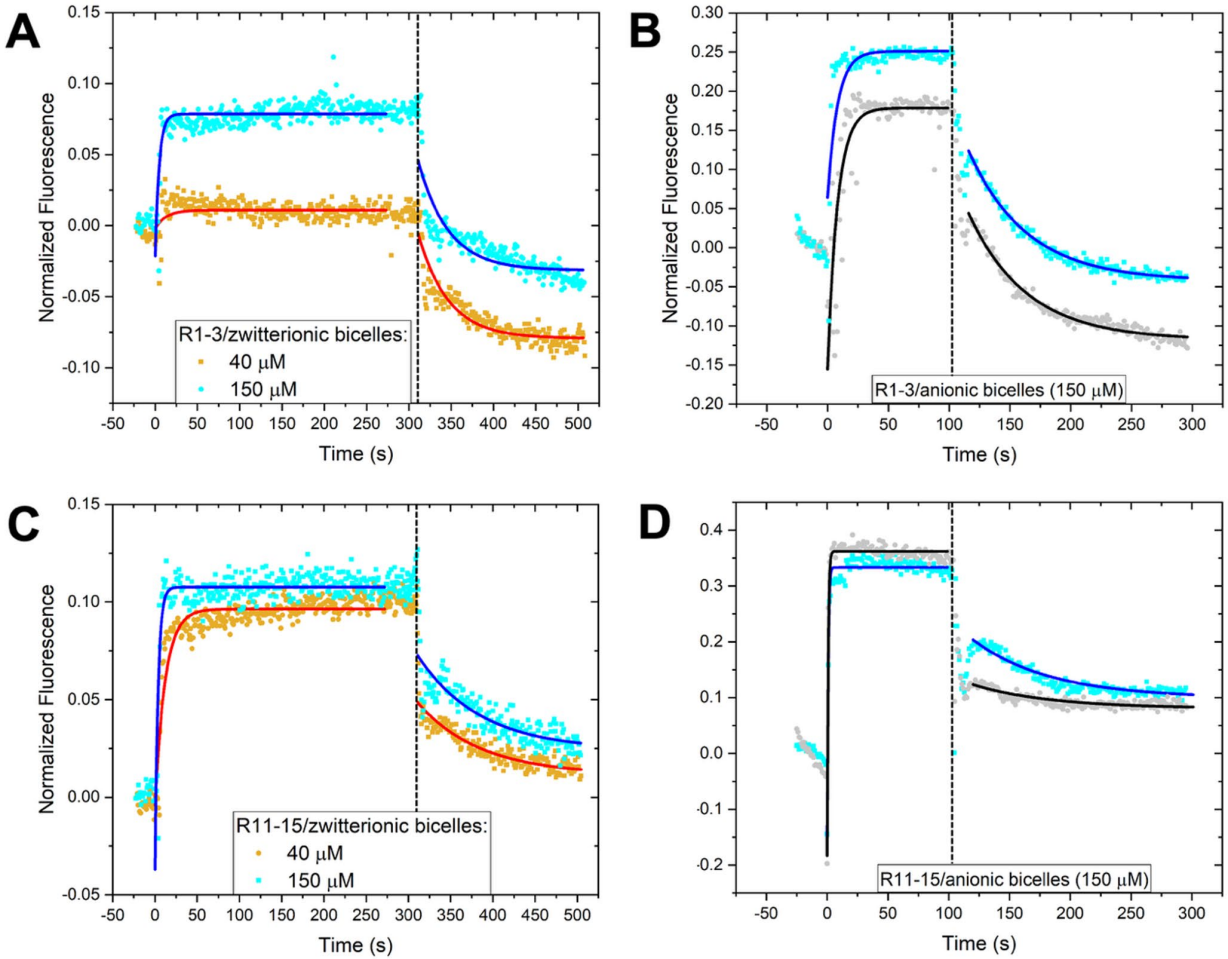
Kinetic analysis of R1-3 and R 1–15 dystrophin fragments interacting with zwitterionic or anionic bicelles. Association and dissociation phases (time in seconds) are shown based on normalized fluorescence, with the two phases separated by a dotted line. Fits (solid lines) are shown over the time intervals of association and dissociation phases. For each panel, data under the two conditions were co-fitted. (**A**) R1-3 fragment with zwitterionic bicelles at 40 µM (orange) and 150 µM (cyan); *R*^2^ = 0.99. (**B**) R1-3 fragment with anionic bicelles at 150 µM, measured in duplicate (cyan and grey); *R*^2^ = 0.97. (**C**) R11-15 fragment with zwitterionic bicelles at 40 µM (orange) and 150 µM (cyan); *R*^2^ = 0.99. (**D**) R11-15 fragment with anionic bicelles at 150 µM, measured in duplicate (cyan and grey); *R*^2^ = 1.00

For the interaction of the R1-3 dystrophin fragment with zwitterionic bicelles, we measured a *K*_*d*_ of 23 ± 3 µM, consistent with the affinity determined by MST (~ 23 µM) (Dos Santos Morais et al. 2018) (Fig. 4A). This relatively weak interaction aligns with the dynamic and reversible membrane-binding behaviour of dystrophin during cycles of muscle contraction and elongation. Measurements with full-length dystrophin were not feasible, as this large protein has not yet been expressed or purified in an intact form.

In comparison, the binding of the R1-3 dystrophin fragment to anionic bicelles showed slightly weaker affinity (*K*_*d*_ = 31 ± 4 µM; Fig. 4B), primarily due to a lower association rate constant (*k*_*on*_ = 6.7 ± 0.8 10^2^ M^− 1^ s^− 1^) relative to zwitterionic bicelles (*k*_*on*_ = 12 ± 1 10^2^ M^− 1^ s^− 1^). Dissociation rate constants differed significantly between bicelles types, although they remained within the same order of magnitude: *k*_*off*_ = 28 ± 2 s^− 1^ for zwitterionic and *k*_*off*_ = 20.9 ± 0.4 s^− 1^ for anionic bicelles.

The R11-15 dystrophin fragment exhibited higher binding affinities to both zwitterionic and anionic bicelles, with *K*_*d*_ of 7.8 ± 0.9 µM and 1.8 ± 0.2 µM respectively, compared to the R1-3 fragment (see above). While the dissociation rates were similar for both bicelles types (*k*_*off*_ = 14 ± 1 s^− 1^ for zwitterionic and *k*_*off*_ = 16 ± 1 s^− 1^ for anionic bicelles), the association rate was significantly higher for anionic bicelles (*k*_*on*_ = 86 ± 4 M^− 1^ s^− 1^) than for zwitterionic bicelles (*k*_*on*_ = 17.2 ± 0.9 M^− 1^ s^− 1^), opposite to the trend observed for R1-3 (Fig. 4, C-D). Consequently, R11-15 associates more rapidly with bicelles and exhibits stronger affinity toward anionic bicelles relative to R1-3 (Table 1).

The higher affinity of R11–15 for bicelles compared to R1-3, likely arises from its extended architecture and enhanced conformational flexibility, which may promote multivalent electrostatic interactions and improved adaptation to membrane curvature and packing defects, thereby maximizing lipid contact.

**Table 1:** Kinetic parameters for the interaction of R1-3 and R11-15 dystrophin fragments with zwitterionic and anionic bicelles using the “bicelle analyte” strategy

	k_on_ (10^2^ M^− 1^ s^− 1^)	k_off_ (10^− 3^ s^− 1^)	K_d_ (µM)
DMPC/DHPC zwitterionic bicelles			
***R1-3***	12 ± 1	28 ± 2	23 ± 3
***R11-15***	17.2 ± 0.9	14 ± 1	7.8 ± 0.9
DMPC/DMPS/DHPC anionic bicelles			
***R1-3***	6.7 ± 0.8	20.9 ± 0.4	31 ± 4
***R11-15***	86 ± 4	16 ± 1	1.8 ± 0.2

The stronger binding observed for R11-15 and anionic bicelles, compared to zwitterionic, likely reflects the contribution of electrostatic interactions mediated by negatively charged lipids, in agreement with the established role of dystrophin in membrane stabilization through charge-dependent mechanisms.

Overall, dystrophin fragments-bicelles interactions display moderate to low affinities, with *K*_*d*_ values ranging from 1.8 to 33 µM depending on bicelle type and surface immobilization strategy. Control experiments using conalbumin immobilized on the chip surface revealed no detectable binding to bicelles, confirming the specificity of the dystrophin-bicelle interaction (Fig. S1A).

Kinetics were also investigated using the dynamic mode of switchSENSE^®^, in which the DNA nanolevers oscillate at kHz frequencies during association and dissociation steps. However, this mode did not allow reliable affinity measurements (data not shown), likely due to the large size of bicelles and/or steric hindrance at the sensor surface.

**Fig. 5:**
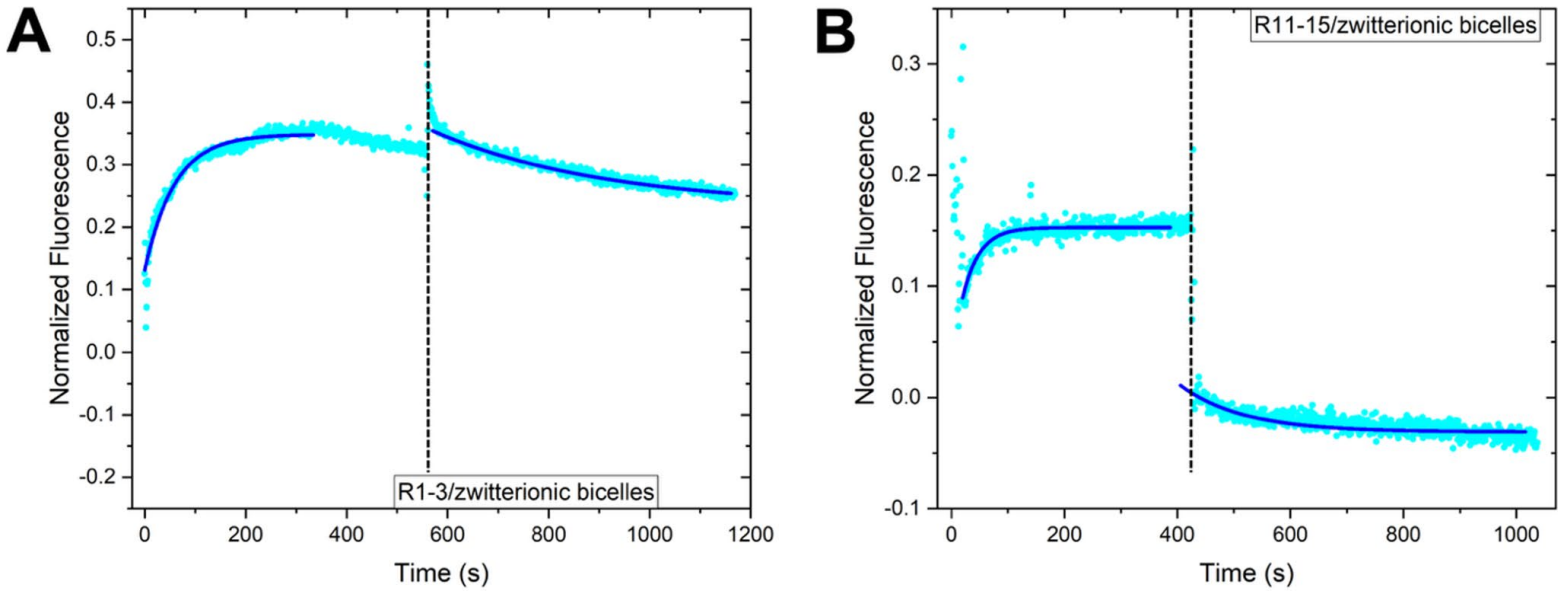
Kinetic analysis of R1-3 and R11-15 dystrophin fragments interacting with zwitterionic bicelles. Association and dissociation phases (time in seconds) are shown based on normalized fluorescence, with the two phases separated by a dotted line. All measurements were performed at 150 µM for both fragments: (**A**) R1-3, *R*^2^ = 1.00; (**B**) R11-15, *R*^2^ = 0.96

***“Dystrophin analyte” strategy***. As a control for the “bicelle analyte” setup, an alternative configuration was employed in which bicelles were immobilized on the surface via cholesterol-modified ssDNA. To ensure consistency with previous measurements and to optimize the accessibility of surface-bound bicelles, 96-DNA NLs were used. The immobilization of bicelles on cholesterol-modified cNLs relies on the hydrophobic insertion of cholesterol into lipid assemblies. This strategy was tested with zwitterionic bicelles to evaluate their interaction with both R1-3 and R11-15 dystrophin fragments (Fig. 5). Successful capture of bicelles on the DNA-cholesterol-functionalized chip was confirmed by an association rate constant of *k*_*on*_ of 4.2 ± 0.1 M^− 1^ s^− 1^ (Fig. S2C). Both dystrophin fragments displayed similar binding behaviour under this configuration, with comparable kinetics parameters and affinities (Table 2).

**Table 2:** Kinetic parameters for the interaction of R1-3 and R11-15 dystrophin fragments and zwitterionic bicelles using the “dystrophin analyte” strategy

	k_on_ (10^2^ M^− 1^ s^− 1^)	k_off_ (10^− 3^ s^− 1^)	K_d_ (µM)
R1-3 dystrophin fragment	0.9 ± 0.1	2.9 ± 0.1	31 ± 2
R11-15 dystrophin fragment	1.8 ± 0.1	6.1 ± 0.6	33 ± 4

The dissociation constant (*K*_*d*_) for the interaction between dystrophin fragment R1-3 and zwitterionic bicelles using the “dystrophin analyte” strategy (31 ± 2 µM) is comparable to that obtained with the “bicelles analyte” approach (23 ± 3 µM). In contrast, for the R11-15 dystrophin fragment, *K*_*d*_ measured using the “dystrophin analyte” strategy (33 ± 2 µM) is approximately fourfold higher than with the “bicelles analyte” approach (7.8 ± 0.9 µM) (Table 1). This difference primarily reflects a markedly lower association rate constant (*k*_*on*_*=*1.8 ± 0.1 10^2^ M^− 1^ s^− 1^) in the “dystrophin analyte” setup compared to the “bicelle analyte” approach (*k*_*on*_*=* 17.2 ± 0.9 10^2^ M^− 1^ s^− 1^), likely due to enhanced accessibility or favourable exposure of binding sites when dystrophin is immobilized rather than in solution. When dystrophin is immobilized, its orientation is constrained but likely favours consistent exposure of the lipid-binding interface, thereby enhancing encounter efficiency. In contrast, when dystrophin is free in solution, transient conformational states, dynamic shielding of interaction surfaces, and diffusion-limited collision geometry may reduce the effective association rate. Thus, the observed differences likely reflect variations in molecular orientation, encounter probability, and diffusion rather than simple accessibility of binding sites.

These results indicate that the “bicelle analyte” strategy, with smaller dystrophin fragments immobilized on the surface, is better suited for detecting weak interactions, albeit requiring higher protein concentrations. The lower affinity observed in the “dystrophin analyte” setup may also be influenced by the less stable attachment of bicelles via cholesterol-modified DNA, compared to covalent crosslinking of dystrophin fragments in the “bicelle analyte” method. Although binding remained generally stable, a slight fluorescence decrease during the association phase was observed (data not shown), likely reflecting partial detachment of bicelles from the surface. As with the “bicelle analyte” strategy, no nonspecific binding was detected between either bicelle type and a control protein (streptavidin), supporting the specificity of dystrophin-bicelle interactions (Fig. S1B).

Furthermore, the distinct behaviours observed between the R1–3 and R11–15 fragments likely arise from differences in their structural organization and charge distribution (Delalande et al. 2018), suggesting that multiple lipid-binding mechanisms contribute to dystrophin’s membrane association. These findings highlight the functional specialization of dystrophin domains and emphasize the relevance of lipid composition in modulating binding kinetics and affinity.

## Conclusions

This study highlights the utility of the switchSENSE^®^ platform for quantitative characterization of weak biomolecular interactions, with dissociation constants (*K*_*d*_) approximately 30 µM. These results position switchSENSE^®^ among the few surface-based biosensor technologies capable of reliably detecting and quantifying low-affinity binding events.

By employing complementary immobilization strategies – where either bicelles or dystrophin fragments served as analytes or ligands – we validated the robustness and reproducibility of kinetic and affinity binding measurements. The technique enabled precise determination of association (*k*_*on*_) and dissociation (*k*_*off*_) rate constants, as well as equilibrium dissociation constants (*K*_*d*_), for interactions between two types of phospholipidic bicelles (zwitterionic and anionic) and two dystrophin fragments (R1-3 and R11-15; Table S1).

Our findings demonstrate that switchSENSE^®^ allows accurate quantification of interactions between a multidomain peripheral membrane protein and membrane-mimicking lipid assemblies. The system provides insights into the dynamic and reversible nature of dystrophin binding to phospholipid surfaces, reflecting the cytoplasmic face of the sarcolemma in muscle cells, and underscore the broader applicability of switchSENSE^®^ for studying transient or low-affinity interactions in membrane-associated protein function.

Notably, the *K*_*d*_ values for R1-3 dystrophin fragment with both zwitterionic and anionic bicelles (20–30 µM range) align with previous reports. Newly determined *k*_*on*_ and *k*_*off*_ values indicate that the low affinity is primarily driven by rapid dissociation. The observed reversibility of dystrophin binding, not previously demonstrated by other techniques, may hold physiological significance in the context of dynamic membrane interactions.

In summary, switchSENSE^®^ provides a powerful, reproducible platform for quantifying protein-lipid interactions, offering a valuable tool for investigating weak and transient biomolecular interactions across diverse biological systems.

## Supplementary Information

Below is the link to the electronic supplementary material.

Supplementary Material 1

## Data Availability

The data that support the findings of this study are available on request from the corresponding author (PFV).
