# Supplementary material for: Weak protein–bicelle binding quantification via surface-based DNA nanolevers
Source: Eur Biophys J. 2026 Mar 2;55(3):379–90. doi: 10.1007/s00249-026-01829-4 (PMC13319134; doi:10.1007/s00249-026-01829-4)
Supplement: Supplementary file 1 — Supplementary Material 1 [file 249_2026_1829_MOESM1_ESM.docx]

**Supplementary Information for:**

**Weak Protein–Bicelle Binding Quantification via Surface-Based DNA Nanolevers**

Sophie COMBET^1*^, Raphael DOS SANTOS MORAIS^1,2^, Audrey COMTE^3^, Angélique CHERON^2^, Emeline BARBET-MASSIN^4,£^, Hanna MÜLLER-LANDAU^4,¥^, Olivier DELALANDE^2^, Jean-François HUBERT^2^, Jean-Baptiste CHARBONNIER^3^, and Paloma Fernández VARELA^3*^

^1^Laboratoire Léon-Brillouin (LLB), UMR12 CEA, CNRS, Université Paris-Saclay, F-91191, Gif-sur-Yvette CEDEX, France

^2^Université de Rennes, CNRS, IGDR, UMR6290, F-35000 Rennes, France

^3^Institute for Integrative Biology of the Cell (I2BC), CEA, CNRS, Université Paris-Saclay, F-91198 Gif-sur-Yvette CEDEX, France

^4^Dynamic Biosensors GmbH, Perchtinger Str. 8/10, D-81379 München, Germany

Current addresses:

^£^JEOL Germany GmbH, Gute Änger 30, D-85356 Freising, Germany

¥Quattro research, Fraunhoferstrasse 18a, D-82152 Planegg-Martinsried, Germany

^*^Corresponding authors:

sophie.combet@cea.fr; ORCID 0000-0002-8672-4514

pfvarela@gmail.com; ORCID 0000-0001-5078-7102

**Negative controls**

To verify the specificity of dystrophin-bicelle interactions, we tested nonspecific proteins known not to interact with lipids. Conalbumin was used with anionic bicelles in the “bicelle analyte” strategy, and streptavidin was used with zwitterionic bicelles in the “dystrophin analyte” strategy (Fig. S1).


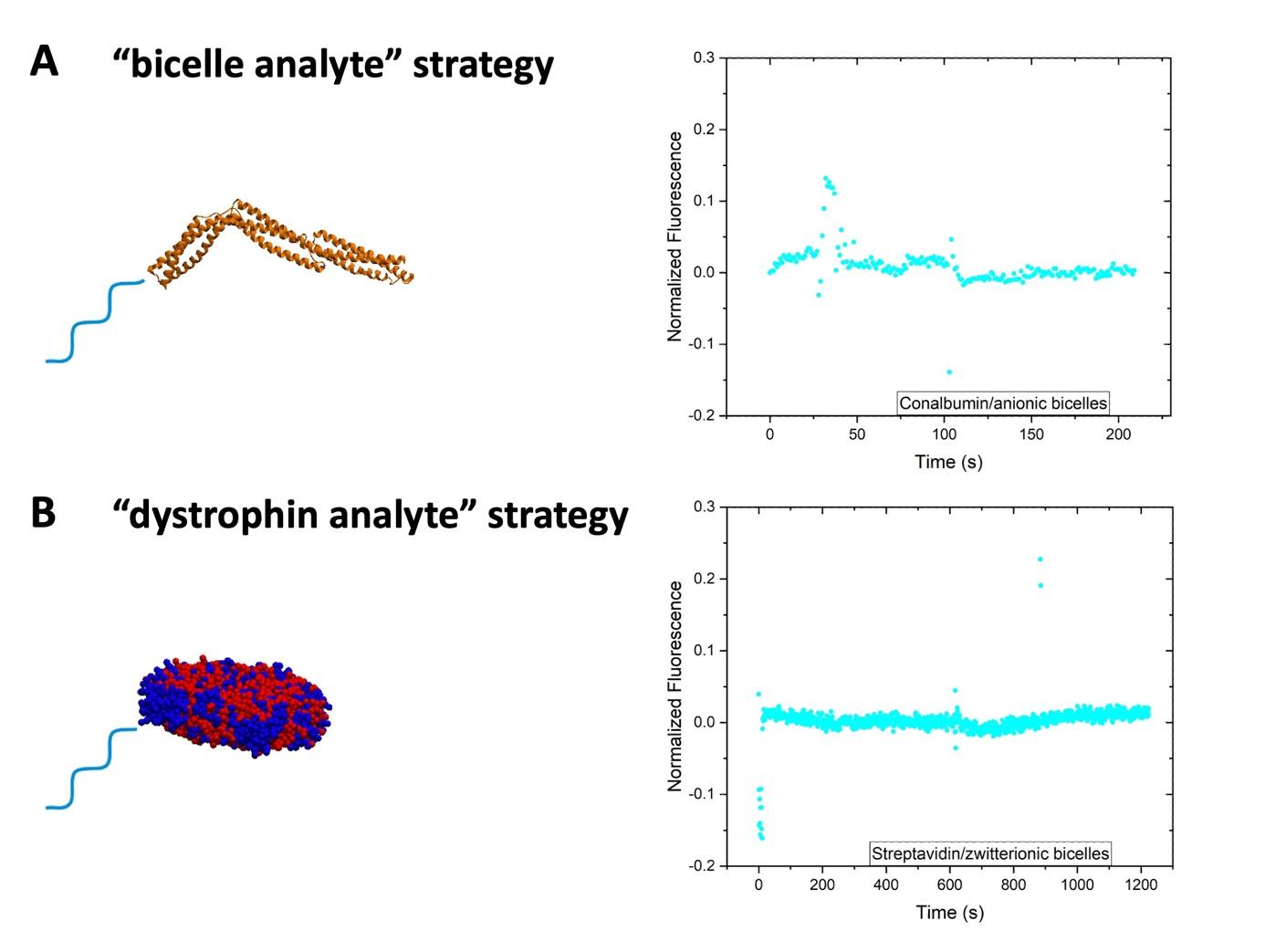


**Figure S1. Negative controls.** Normalized fluorescence during the association phase is shown. Two strategies were employed: (**A**) anionic bicelles used as analytes with 150 µM conalbumin as a negative control, showing no detectable interaction; (**B**) immobilized zwitterionic bicelles probed with 150 µM streptavidin as analyte, also showing no interaction. These results confirm the specificity of the assay.

**Table S1. Summary of all measured kinetic values between bicelles and dystrophin fragments (compiled from Tables 1 and 2 in the main article).**

|  | ***k_on_* (10^2^ M^− 1^ s^− 1^)** | ***k_off_* (10^− 3^ s^− 1^)** | ***K_d_* (µM)** |
| --- | --- | --- | --- |
| ***“bicelle analyte” strategy*** | | | |
| *DMPC/DHPC* ***zwitterionic*** *bicelles* | | | |
| ***R1-3*** | 12 ± 1 | 28 ± 2 | 23 ± 3 |
| ***R11-15*** | 17.2 ± 0.9 | 14 ± 1 | 7.8 ± 0.9 |
| *DMPC/DMPS/DHPC* ***anionic*** *bicelles* | | | |
| ***R1-3*** | 6.7 ± 0.8 | 20.9 ± 0.4 | 31 ± 4 |
| ***R11-15*** | 86 ± 4 | 16 ± 1 | 1.8 ± 0.2 |
| ***“dystrophin analyte” strategy*** | | | |
| *DMPC/DHPC* ***zwitterionic*** *bicelles* | | | |
| ***R1-3*** | 0.9 ± 0.1 | 2.9 ± 0.1 | 31 ± 2 |
| ***R11-15*** | 1.8 ± 0.1 | 6.1 ± 0.8 | 33 ± 4 |


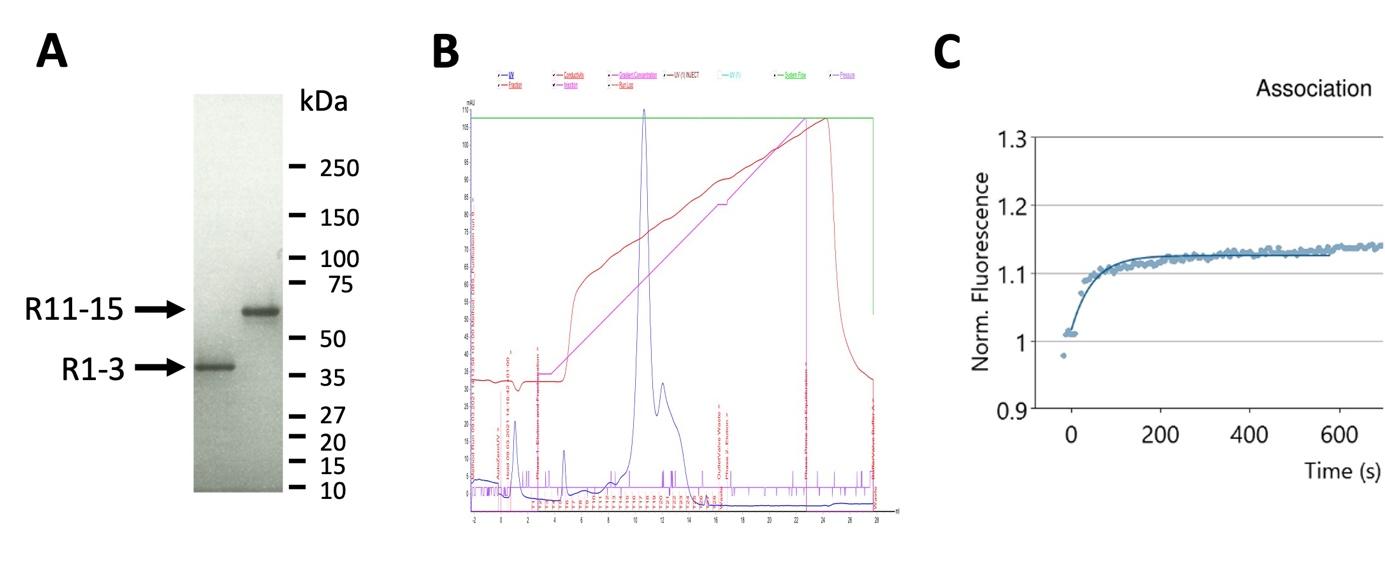


**Figure S2. switchSENSE® sample preparation.** (**A**) SDS-PAGE analysis of R1-3 and R11-15 dystrophin fragments. (**B**) Purification of DNA-crosslinked R1-3 dystrophin fragment by anion exchange chromatography. (**C**) Immobilization of zwitterionic bicelles on DNA-cholesterol-functionalized chips.
